# Treatment of Fracture of the Jaw, with Critical Remarks

**Published:** 1880-08

**Authors:** Thomas Brian Gunning

**Affiliations:** New York


### The Practitioner.

Vol. I.——*August, 1880.*——No. 8.

ARTICLE I. PART III.

### TREATMENT OF FRACTURE OF THE JAW, WITH CRITICAL REMARKS.

BY THOMAS BRIAN GUNNING, D. D. S., NEW YORK.

It is necessary to bear in mind that the *position* of the lower jaw is *peculiarly* dependent upon the muscles attached to it. Neglect of this has caused great mistakes, both in diagnosis and treatment; patients having been put to much suffering by the endeavors of surgeons to set fractures which did not exist; the displacements supposed to indicate them being the result of fracture in another part of the jaw (the latter being drawn out of shape by the muscles through laceration, contusion or severe swelling) and *thereby prevented from going into proper atriculation with the upper jaw*, while the surgeon supposed that the ramus or neck of the condyle was broken.* This also occurs when there is no fracture of the bone.

With only incomplete fracture, in which the bone retains its shape so perfectly that treatment is unnecessary, weeks or even months may elapse before the muscles are able to bring the jaw into place so that the lower teeth will close against the upper as before the injury. In fact, this inability may be present *without any* fracture of the bone.

Malgaigne† maintains that muscular action cannot of itself induce displacement, even in cases where the violence has dis-connected the fragments by tearing the periosteum and the surrounding tissues. This is opposed to the views of other observers, and is disproved by the action of the muscles. M. Malgaigne's position made it necessary to notice his radical mistake at this time. Cases 4, 6, 7 and 8

*See case 4, which shows the effect of a cut in the masseter muscle.

† Malgaigne, op. cit.

show conclusively the influence of the muscles and confirm the views of their action as explained in the preceding pages. Other cases treated and seen by me also demonstrate that the opinion expressed so decidedly by Malgaigne and entertained by other writers, as to the effect of the impulse given by the cause of fracture upon displacement, is erroneous. For the impulse being exhausted in deciding the position, direction and extent of the injury to the bone and surrounding tissues, the bone is then *surrendered* to the muscles which affected it before and at the time of fracture, and still continue to do so, according to the *condition in which it and they are left.*

If pain and swelling, or either, set in after the jaw has been subjected to a shock, to a blow, or to compression, it may still be unfractured; reliable details as to how the injury was received in connection with the age and condition of the patient, are quite important, but to all this there must be superadded careful examination before the nature of the injury can be known. When the surrounding tissues, periosteum, gum, &c., are torn by the separation of the fragments of the bone, the muscular displacement is likely to be great, unless, as sometimes seen, the fragments are, by the impact which broke the bone, driven into such positions as to remain in contact in opposition to the muscles; *the displacement of the accident being maintained.* This impacted condition of the fragments is, however, rarely seen.

The fracture of the bone is generally *compound*; that is, the broken bone is through a tear, cut or other opening in its investing tissues exposed to the air, this being mostly the case in fracture of the body, as the fibrous coat of the periosteum tears readily and the mucous covering is very thin; but the bone is seldom exposed though the external parts are frequently contused.

The muscular displacement will be proportioned to the separation of the parts at the time of fracture, even if the fracture is *simple*, that is, without any tear of the periosteum and investing tissues, the effect of the muscles is present, although it may not always be apparent.

If the anatomy of the parts involved and the muscular action which controls and effects the lower jaw were well understood, very little need be said at this time; unfortunately, however, some who undertake to instruct as clinical surgeons, or as authors of formal treatises, fail to set the matter correctly before the student.

When the body of the jaw, which supports the hyoid bone and its appendages, is fractured vertically in or near the front, the shorter fragment will rise if the muscles are unhurt, and this even if the fracture is at the upper border precisely over the symphysis and only shortens one side by passing down at its expense. In the first instance the longer fragment bears most of the weight of the larynx and trachea, and the shorter one will be drawn up by the muscles which pass down from the skull to the ramus, and in the second the short fragment will also ride up to the slant of the other, and in both cases the teeth will show it. These conditions will be more marked in proportion as the one fragment is longer than the other, but should the fracture pass down at the expense of the longer fragment, the teeth may be level, though perhaps a little separated, as the shorter fragment may by its projecting end support the longer. If the fracture is in the thickness of the bone, that is as if the break should commence in the outer line of the symphysis and pass back on one side so as to come out near the canine tooth, which is perhaps more frequently seen near the angle at the back of the teeth, or in parts where they have been lost, the short fragment is generally outside. This was so in case 4 in which the fracture was in the left lateral socket and passed back on the outside of the left fragment. In case 5 however, in which the break was in the body behind the right canine and passed down in the depth of the bone at the expense of the back fragment its sharp, upper, bare edge pointed inward to the symphysis. The muscular action was consistent in both instances, the difference in the positions being the result of the direction of the fractures, and the sound in the left masseter of case 4.

Fracture in and near the angle, or in the ascending ramus is readily detected, according to my own observation they are apt to be very painful. I cannot agree with Professor D. Hayes Agnew, who says:* "When the ramus of the lower maxilla is the part implicated, there is generally not much disarrangement of the fragments, in consequence of the two planes of muscles, the masseter and the pterygoid, covering the part of the bone, and acting as splints." The internal pterygoid muscle passes from its origin so much outward to reach its insertion on the angle that the *external pterygoid* separates it from the upper part of the ramus; its action is therefore likely to draw the angle in if this is broken clear of the body. In case 4, this displacement would have probably resulted had the jaw been broken where

* The Principles and Practice of Surgery, vol. 1, p. 843; J. B. Lippincott & Co., Philadelphia, 1878.

the masseter was cut so deeply. In case 7 the bone was so split that the inside was a part of the body, and kept the angle out. If fractures in the *ramus* is seen early or when there is little swelling it may be often felt in the mouth. When looking along the range of the teeth and no indication of fracture is seen in the body of the jaw, if the finger is laid against the outside of the back tooth, its end will feel the anterior edge of the ramus which, when the mouth is opened wide may be explored up to the sharp edge of the *coronoid process.* Any displacement along this ascending border could be felt, unless the parts were swollen, and if so, crepitus and pain on proper manipulation, would indicate the seat of the fracture which in any part of the ascending ramus would generally not be on the same side with a fracture in the horizontal body of the jaw.

Professor Agnew also says: *"Fracture of the *coronoid* process is an accident of great rarity and is difficult to diagnose. It may be conjectured when a finger placed over the temporal region fails to detect any movement of the temporal muscle when the mouth is opened and closed."

From my own experience it is likely that the *coronoid* process is broken from the ramus as frequently as the *condyle.* Its fracture, however, interferes with the use of the jaw so very little, compared with a break in the neck of the condyle, that it easily passes unnoticed, especially as it probably occurs only when the jaw is broken on the other side.

The blow or pressure which falls upon one side of the jaw and breaks it, may easily drive it over to the other side and fracture the coronoid process against the inside of the malar bone.

Attention has been called to the facility afforded by the mouth to examine the anterior border of the ramus, which commences with the upward curve of the exterior oblique line near the outer side of the back tooth. Now if the finger is turned down over the inside of this tooth so as to reach under the *internal* oblique ridge, it will be felt that the alveolar process ends very abruptly, both the basilar portion of the body, and the ramus of the jaw, passing outward, and leaving the sockets of the teeth overhanging and projecting in. It follows that when a fracture passes through the socket of the back tooth, it as in case 7 before referred to, is apt to leave the outer side of the ramus whole, that is, break the body and ramus apart, except, per-

*Op. Cit., Vol. I p. 843.

haps, a scale of the inner surface of the ramus, which may separate as far back as the mylo-hyoid groove, or even up to the inferior dental foramen, and project from the body while the ramus retains a part of the external oblique, at the expense of the outside of the body, this sharp portion frequently rising into the tissues and causing much pain.

When, however, the back tooth has been lost so long that the socket is filled up, the bone is very strong at this part, and fracture in the ramus is more likely to be found higher up, and across the bone, rather than splitting it. In which case, the anterior border will be the most likely place to find displacement, for even admitting that the masseter muscle should, as suggested by Professor Agnew, act as a splint, still there is the external pterygoid to pull the upper part of the ramus forward, and the muscles attached to the body of the jaw to pull the lower part of the ramus backward, and it is hardly possible that the anterior border should remain from the displacement and pain, even if not displaced laterally. In fact, I have seen in both sexes, and with the back teeth present, fracture in the ramus which passed back through the bone so that the portion below was separated from the part holding the condyle and the coronoid process.

Now, of the precise direction in which the fracture passes back in the ramus, no exploration can be made back of the rounded, upward continuation of the internal oblique ridge, as beyond this, the palate and superior constrictor muscles with the tonsil, &c., block the way. The direction of the fracture may, therefore, be looked for outside of the mouth.

The external surface of the ramus gives attachment to the masseter muscle, and the internal surface at the angle and at the front part respectively to the internal pterygoid and the temporal muscles, but there is a large central portion weakened by the inferior dental foramen and canal which is not protected by muscular adhesion, and a blow on the outside may not only fracture the ramus but also displace the fragments.

In examination of the external surface, the masseter muscle is in the way but it does not reach to the posterior border of the ramus, except at the angle, from which up, the portion of the bone covered only by the skin, etc., gradually widens, so that the condyle and the ramus also, is free of muscular adhesion to the centre of the sigmoid notch.* Thus the posterior border which is clear of muscular adhesion, affords the best place for examination outside the mouth, and. as before shown, the anterior border inside. This comparatively uncovered condition of this condyle is important.

It has been seen that the condyle moves forward and the angle backward to depress the jaw and open the mouth, but it is now advisable to closer attention to the relations of the condyle. Its movement is clearly felt if the finger is pressed into the ear. With the teeth of both jaws closed against each other, the finger, if placed in front of the ear, will rest upon the condyle, and if this is drawn forward by opening the teeth, the finger will sink into the hollow left behind, or with the finger placed under the ear it will at once be felt that the part of the ramus then on a level with it, neither moves backward nor forward.

Now with one hand taking firm hold of the jaw, by means of the teeth and chin, while the fingers of the other are on or near the condyle, it could be felt whether movement of the body of the jaw, take the condyle with it, if it does, the bone is sound; if it does not, crepitus, pain and displacement will indicate the seat of fracture. The natural motions of the jaw in opening and shutting, and in the forward and lateral movements, as in cutting with the front teeth and in grinding the food, also give, when intelligently studied, clear indication of the position and character of the injury.

This displacement caused by any muscular action is of course modified by the direction of the fracture and the condition of the surrounding tissues. The facility afforded by the open jaw to examine the anterior border of the ramus even up over the curve of the coronoid process is little more than is afforded by opening the lips only when the jaws are shut together, in regard to injury below the coronoid process. The fracture of this process, as indicated in the mouth, having been remarked upon, we now pass to its external symptoms, as these also require examination in connection with those indicating fracture in other parts of the bone, especially in the ramus.

The cheek affords a good place for examination, for when the mouth is open the point of the coronoid process is somewhat below the border of the malar process, and with a finger on each side, comparison between the suspected coronoid process and the opposite one, would indicate more clearly than the finger over the *temporal region.*

*The parotid gland enfolds the neck of the condyle, but need not be considered here.

The *masseter* muscle will, it is true, tighten somewhat when the jaw is depressed, but this will not mislead so much as the movement of the temporal.

Professor Agnew is mistaken in supposing that if the coronoid process is fractured, the temporal muscle is still when the mouth is opened and closed. In health, movement of this muscle may be as distinctly felt in opening the jaw as in closing it, and this can be felt also when the coronoid process is fractured. If the jaw cannot be moved, still examination on or near the coronoid process would be more likely to lead to correct understanding of the injury than if made over the temporal region. I present one case in illustration:

On May 28th, 1864, a boy eleven years old was crushed by a pile of lumber falling on him, and received two fractures in the thigh, one in the left clavicle, and one in the lower jaw between the left canine and bicuspid teeth, while there was much pain and swelling in front of the right ear and along under the zygomatic and malar processes.

The injury was six days old when I first saw it by the request of Dr. Freeman, and the jaw moving pretty well on the right side. I decided to leave it free to move during the treatment of the fracture on the left side.

The splint applied was the original of fig. 4. He used it in eating from the time it was first put on, and in six weeks from the injury his jaw was well, and the splint left off, but the coronoid process had not united, the corner left by the fracture on the ramus being felt, but the coronoid process was gone.

The boy had lost his mother, and his father being absent all day he by screaming, compelled his sisters to keep him supplied with crackers, which he crushed between the top of the splint and his upper teeth.

The temporal muscle is inserted into the ramus as well as into the coronoid process or his jaw would have been more crippled.

This case shows conclusively that fracture of the coronoid process is not indicated by the temporal muscle, also that the inside of the mouth is the best place to look for injury of this process.

Hamilton, without having seen a fracture of this process, says: "It is probable that an examination by the finger within the mouth, would readily detect mobility and displacement."

The persistent action of the temporal muscle is equalled by its antagonist, the *External Pterygoid.*

With recent fracture in the body, the angle or the ramus, or in any part below the neck of the jaw, and in injuries where any of these parts are absent, the *external pterygoid* at ouce pulls the condyle and cartilage forward when the jaws are separated. The pain of recent injury may prevent movement, but I do not recollect an instance in which the *external pterygoid* was in fault.

In one case, through injury from a shot, the left angle and ramus above, was destroyed, except the condyle from which a short spine of the back border of the ramus still projected downward; these being in substitution of the bone, lost only a fibrous connection between this spine and neck of the condyle and the body of the jaw. The condyle, however, was in its normal position and came forward with its fellow of the other side to open the jaw. Now, in this case the ball which passed through the ramus shattered it so much that the necrosed pieces which came away left the condyle and its interarticular cartilage under control of the external pterygoid, without any part of the ramus to interfere with it. The result confirmed my explanation of the muscular action which depresses the lower jaw and opens the mouth, while it confutes the statements made respecting the effect of the external pterygoid upon the condyle, when its neck is fractured.

In respect to fracture of the neck of the jaw, Malgaigne says: "This fracture is exceedingly rare, and for my own part I have never seen it. It appears to have been observed by Soranus, after which no more mention of it occurs until the time of Desault, and I know at present of but eight published instances."*

Malgaigne, after referring to the fact pointed out by Ribes—that in laxation of the condyle, the chin is carried to the opposite side, while in fracture of its neck the chin deviated to the same side, says: "The displacement of the condyle, induced by the cause of the fracture, increased and maintained by the action of the external pterygoid muscle, is a main point in the history of this fracture. The condyle itself remains in relation with the glenoid cavity, but the pterygoid muscle makes it execute a movement of rotation, carrying the fractured neck upward, forward and inward, so that the fractured surface of the inferior fragment is in relation only with the posterior surface of the neck and of the condyle."

"The diagnosis is generally easily deduced from the symptoms

* Packard's edition of Malgaigne, vol. 1. p. 342.

pointed out; we can, moreover, explore the fracture at once, externally and internally, by passing a finger into the mouth." *

Professor Frank Hastings Hamilton, whose treatise appeared one or two years after Dr. Packard's translation of Malgaigne, in speaking upon the reduction of the displaced condyle by Ribes, says: "The case of a cannonier whose jaw was broken through the neck of the condyle on the right side, and through its body on the left, afforded him an opportunity to determine the practicability of a method of which he had as yet only conceived the idea. Malgaigne thus describes his procedure. "With the left hand seize the anterior portion of the jaw, for the purpose of drawing it horizontally forwards, while you carry the index finger of the right hand to the lateral and superior part of the pharynx. You will meet at first the projection formed by the styloid process, but moving your finger forwards you will find soon the posterior border of the ramus of the jaw; and following this border from below upwards, you will arrive at the inner side of the condyle, which you will push outwards in such a manner as to engage it upon the other fragment.

"This manœuvre cannot be made without causing nausea, as the finger always does when carried into the posterior part of pharynx; but this is a slight inconvenience. The reduction obtained, bear the jaw upwards and backwards in order to press and fix the condyle between it and the glenoid cavity, then fasten it in place with the sling." The fragments were thus early brought into apposition in the case reported by Ribes, and the patient was cured without any deformity."†

*CONCLUDED IN OUR NEXT ISSUE.*

* Packard's edition of Malgaigne, vol. 1, p. 324.

†F. H. Hamilton: Fractures and Dislocation, second edition, p. 129.

